# Non-Invasive Measurement of the Internal Pressure of a Pressurized Biological Compartment Using Lamb Waves

**DOI:** 10.1109/TBME.2021.3129652

**Published:** 2022-05-19

**Authors:** David P. Rosen, Nicholas B. Larson, Azra Alizad, Mostafa Fatemi

**Affiliations:** Department of Physiology and Biomedical Engineering, Mayo Clinic College of Medicine and Science, USA.; Department of Quantitative Health Sciences, Mayo Clinic College of Medicine and Science, USA.; Department of Radiology, Mayo Clinic College of Medicine and Science, USA.; Department of Physiology and Biomedical Engineering, Mayo Clinic College of Medicine and Science, Rochester, MN 55905 USA.

**Keywords:** Pressure measurement, pressure-vessel, ultrasound, Lamb wave speed, bladder

## Abstract

In this study, we propose a mechanical analysis for estimating the internal pressure of a finitely deformed spherical compartment from Lamb wave measurements. The proposed analysis produces a dispersion relation associating Lamb wave speed with pressure using limited material parameters (only a strain stiffening term). The analysis was validated on ultrasound bladder vibrometry (UBV) experiments collected from 9 *ex vivo* porcine bladders before and after formalin cross-linking. Estimated pressures were compared with pressures measured directly by a pressure transducer. The proposed analysis proved broadly effective at estimating pressure from UBV based Lamb wave without calibration as demonstrated by the observed concordance between estimated and measured pressures (Lin’s CCC = 0.82 (0.66–0.91). Theoretical limitations and potential refinements to improve the accuracy and generality of the approach are discussed.

## I. Introduction

Biological compartments that sustain internal fluid pressure are ubiquitous structures in mammalian organisms. Indeed, several allometric scaling relationships observed in hollow organs across mammalian and avian species, such as the isometric scaling observed between organ mass and pressurization energy, can be understood in relation to their mechanical role as pressurized compartments [1]. In relation to human health and medicine, the pressures that such compartments are exposed to are often vital indicators of their functioning. Examples where pressure measurements hold clinical significance in biological compartments include the diaphragm and lungs [2], large blood vessels [3], the heart [4], the bladder [5], [6] and body cavities [7]. In the human bladder, for instance, the pressure across the bladder wall (i.e. detrusor pressure) at end filling can vary from 11.05 ± 5.23 mmHg in healthy bladders to 35.79 ± 70.50 mmHg in non-compliant bladders [8]. Pressure measurements are typically collected by invasive procedures that use catheters instrumented with pressure transducers. Because the potential for discomfort and morbidity is high in such procedures, there is great interest toward non-invasive assessments [3], [9]–[11].

Due to the sensitivity of shear wave speed to tissue loading [12], wave-based elastographic techniques (i.e. shear wave and Lamb wave elastography) are increasingly being investigated as a noninvasive means of inferring loads sustained by soft tissues [13], [14]. Recently, a variety of investigations have correlated internal fluid pressures with transverse wave speeds at the walls of a variety of biological compartments [15]–[19]. However, these investigations are empirical and the underlying mechanics that produce such associations has received limited treatment. An analysis that relates elastic wave speed to pressure in such structures would need to incorporate analyses for: acoustoelastic wave propogation in the presence of finite deformation in a nonlinear and hyperelastic solid [20], [21]; wave dispersion associated with the wall thickness of the compartment [22]; and pressure-vessel analysis for highly deformed compartments [23], [24]. Although previous works have incorporated some components of the described analysis [25]–[28], to the authors’ knowledge, the full combination has yet to be applied toward estimation of pressure in a biological compartment.

In this investigation, we propose a mechanical analysis that relates Lamb wave speed measurement directly to the fluid pressure sustained by a spherical, non-linearly elastic biological compartment. Our approach builds off of the acoustoelastic Lamb wave analysis derived in [25] to develop a dispersion relation that is dependent on pressure, deformation and a single material parameter which is a strain-stiffening term. We couple the aforementioned spherical pressure-vessel analysis to produce a dispersion relation that describes wave speed as a function of pressure. The proposed analysis was validated on ultrasound bladder vibrometry (UBV) measurements collected from *ex vivo* porcine bladders. We applied the analysis to measurements collected before and after a formalin cross-linking treatment was applied to the bladders in order to investigate the pressure estimation in compartments with a wider elasticity range and varied elastic responses to filling.

## II. Theory

**Fig. 1.**
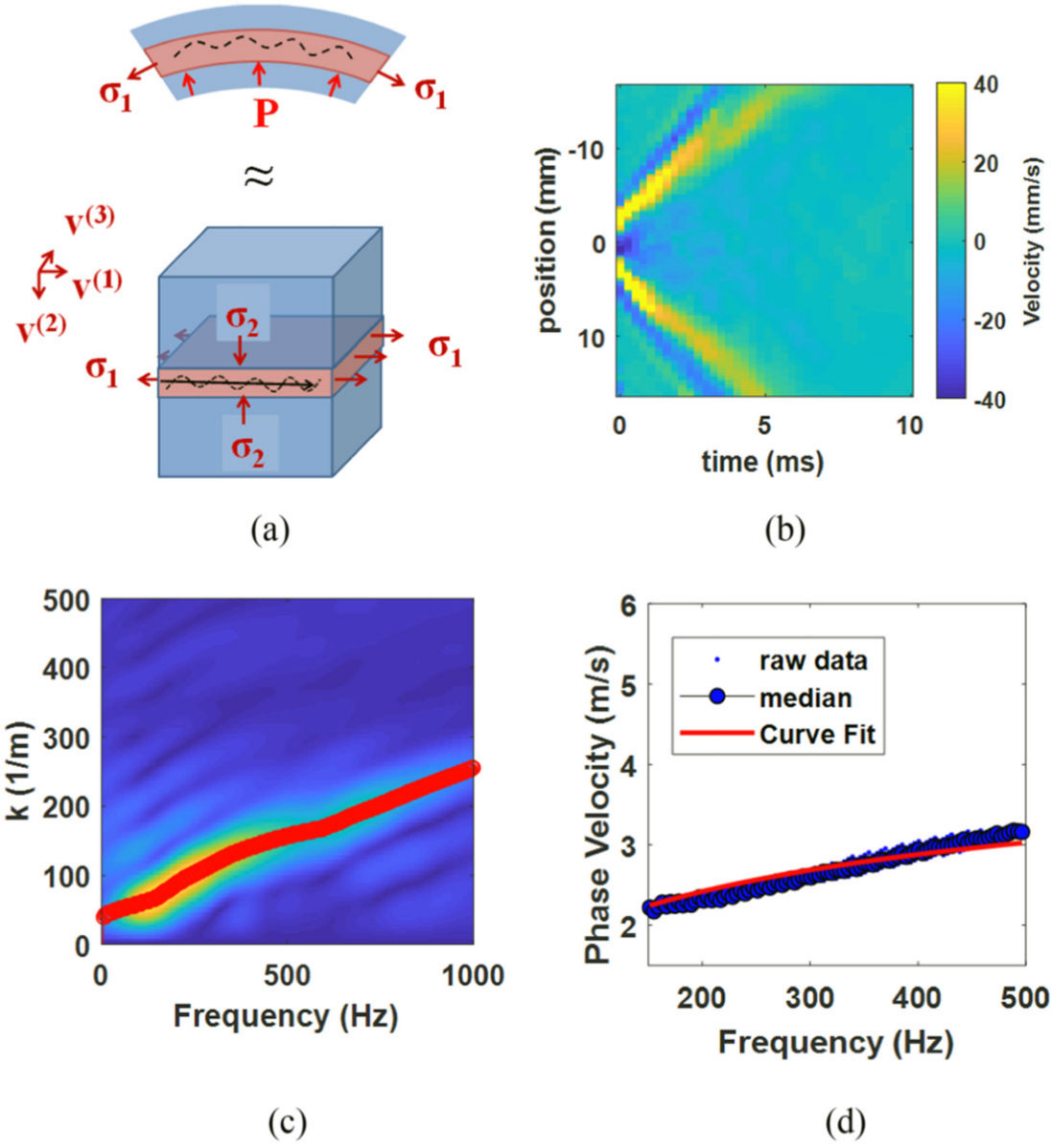
Illustration and example of the analyses applied to the pressurized biological compartment. (a) The compartment is modeled as a hyperelastic spherical pressure-vessel with Lamb waves propagating along the circumference. (b) The acoustic pulse produces Lamb waves propagating forward and backward away from the pulse. (c) A 2D-FFT based method is used to estimate frequency dependent phase velocities [29] from both waves (only a single wave shown). (d) Curve fitting is then applied to the median wave speeds from the 5 acquisitions collected at each volume.

The proceeding sections lay out the mechanical analysis that will be used to relate UBV measurements to pressure measurements collected from *ex vivo* pig bladders. The analysis is broken into three subsections: a concise description of the stress and deformation measures necessary for the analysis (Section II-A); a mechanical treatment relating compartment wall stress to compartment pressure (Section II-B); and an acoustoelastic treatment of Lamb wave propagation in the presence of large deformation due to compartment wall stress (Section II-C). Fig. 1(a) illustrates the system being modeled by the proposed approach.

### A. Stress and Deformation

Preliminary to the proceeding mechanical analysis are expressions of stress and deformation in terms of principal stress and stretches. The left Cauchy-Green deformation tensor and its spectral decomposition are

(1) $$B=FF^{T}=\sum_{i=1}^{3}\lambda_{i}^{2}v^{\left(i\right)}⊗v^{\left(i\right)}$$

Here **F** is the deformation tensor and λ_*i*_ and **v**^(*i*)^ are the principal stretches and principle vectors of **B**. In the case of the pressurized spherical compartment considered here (as illustrated in Fig. 1(a)), the componets of **v**^(*i*)^ align along and across the compartment wall. For an isotropic material, the Cauchy stress (***t***) is co-axial with **B** [30], [31]. As such, the principal Cauchy stress **t** can be expressed relative to λ_*i*_ and **v**^(*i*)^ and a strain energy function *W*. With the inclusion of the incompressibility constraint, it can be expressed as follows:

(2) $$t=\sum_{i=1}^{3}\lambda_{i}\frac{\partial W}{\partial \lambda_{i}}v^{\left(i\right)}⊗v^{\left(i\right)}-pI$$

here, *p* is a Lagrangian multiplier added to enforce the incompressibility constraint.

### B. Spherical Pressure-Vessel Model of the Bladder

In order to relate wall stresses to compartment pressure, the biological compartment is modeled as a thin-walled spherical pressure-vessel subject to some inflation pressure *P*. Without yet imposing the assumption of a thin-wall, the inflation pressure can be expressed in terms of the in-plane principal Cauchy stresses and the inner (*a*) and outer (*b*) radii of the spherical compartment by the following integral [23], [24], [31]

(3) $$P=P_{a}-P_{b}=\int_{a}^{b}\frac{2}{s}\left(t_{1}(s)-t_{2}(s)\right)ds$$

Here *P*_*a*_ and *P*_*b*_ are the absolute pressures at the interior and exterior of the spherical compartment. A key simplifying assumption required for the subsequent Lamb wave analysis is that the principal stretches along the wall of the compartment, and as a consequence of (2) the principle stresses, can be treated as approximately homogenous (i.e. $t_{1}(s)-t_{2}(s)\approx t_{1}-t_{2}$). Applying this assumption to (3) produces

(4) $$P=\left(t_{1}-t_{2}\right)\int_{a}^{b}\frac{2}{s}ds=2\left(t_{1}-t_{2}\right)\ln\left(\frac{b}{a}\right)$$

Approximating (4) by Taylor series expansion to the first order about *b/a* = 1 and replacing *a* and *b* with a single radius *r* and half-thickness *h* via *a* = *r* and *b* = *r* + 2*h* produces

(5) $$P\approx 2\left(t_{1}-t_{2}\right)\left(\frac{b}{a}-1\right)=\left(t_{1}-t_{2}\right)\frac{4h}{r}$$

This is equivalent to the classic wall stress relationship for a thin-walled pressure-vessel [32]. As such, assuming a homogenous stress is intrinsically connected to assuming a thin-walled spherical pressure vessel.

In the case of incompressibility, the principal stretches can be readily defined by the compartment radius *r* relative to its initial value *r*_0_ [23]. They can then, in turn, be determined from the initial and deformed volumes *V* and *V*_0_ via $r={\left(3/4V/\pi \right)}^{1/3}$. These relations are

(6) $$\lambda_{1}=\frac{r}{r_{0}}={\left(\frac{V}{V_{0}}\right)}^{1/3}$$

(7) $$\lambda_{2}=\frac{r_{0}^{2}}{r^{2}}={\left(\frac{V_{0}}{V}\right)}^{2/3}$$

Practically, it is challenging to directly determine an appropriate value for *V*_0_ for soft solids, as it should correspond to the point at which the compartment wall is taut (i.e. is not collapsed), but before the compartment has sustained pressure. A more tractable alternative is to infer *V*_0_ from an undeformed half-thickness *h*_0_ and noting that *h/h*_0_ = λ_2_. As such, rearranging (7) produces

(8) $$V_{0}={\left(\frac{h}{h_{0}}\right)}^{3/2}V$$

### C. Lamb Wave Propogation in a Pre-Stressed Plate

The starting point for the Lamb wave analysis is the result from [25], which derived an expression for Lamb waves propagating in a pre-stressed, isotropic, incompressible and hyperelastic plate surrounded by fluid. There they produced the following dispersion equation for axisymmetric transverse waves propogating in such a condition

(9) $$\begin{array}{l} \gamma s_{1}{\left(1+s_{2}^{2}\right)}^{2}\tanh\left(s_{1}kh\right)-\\ \gamma s_{2}{\left(1+s_{1}^{2}\right)}^{2}\tanh\left(s_{2}kh\right)+\frac{\rho^{F}c^{2}}{\xi }\left(s_{1}^{2}-s_{2}^{2}\right)=0 \end{array}$$

where *h* is half the thickness of the deformed hyperelastic plate (i.e. *h* = λ_2_*h*_0_), *c* is the wave speed, *ρ*^*F*^ is the mass density of the fluid (assumed to be 1 g/ml). *ξ* is a parameter defined as $\xi =\sqrt{1-\frac{c^{2}}{c_{p}^{2}}}$ where *c* is the speed of the transverse wave and *c*_*p*_ is the speed of sound in the fluid (assumed to be 1480 m/s in this current analysis). *k* is the wave number and it defines the frequency dependence of the wave speed through the relation k=2πf/c. $s_{1}^{2}$ and $s_{2}^{2}$ are roots of the quadratic equation defined by

(10) $$\gamma s^{4}-\left(2\beta -\rho c^{2}\right)s^{2}+\alpha -\rho c^{2}=0$$

Here *ρ* is the mass density of the solid wall (also assumed to be 1 g/ml) and *γ*, *α* and *β* are the acoustoelastic parameters for a pre-stressed hyperelastic solid derived by [30]. They are defined by the components of the incremental elasticity tensor $A_{0piqj}=F_{pr}F_{qs}\left(\partial^{2}W/\partial F_{ir}\partial F_{js}\right)$. Using the relationships derived by [30] between $A_{0piqj}$, *W* and λ_*i*_, [25] produced the following expression for *α*, *β* and *γ*

(11) $$\alpha =A_{01212}=\frac{\lambda_{1}^{2}\left(\lambda_{1}\frac{\partial W}{\partial \lambda_{1}}-\lambda_{2}\frac{\partial W}{\partial \lambda_{2}}\right)}{\lambda_{1}^{2}-\lambda_{2}^{2}}$$

(12) $$\gamma =A_{02121}=\frac{\lambda_{2}^{2}\left(\lambda_{1}\frac{\partial W}{\partial \lambda_{1}}-\lambda_{2}\frac{\partial W}{\partial \lambda_{2}}\right)}{\lambda_{1}^{2}-\lambda_{2}^{2}}$$

(13) $$2\beta =A_{01111}+A_{02222}-2A_{01122}-2A_{01221}\;\;=\lambda_{1}^{2}\frac{\partial^{2}W}{\partial \lambda_{1}^{2}}+\lambda_{2}^{2}\frac{\partial^{2}W}{\partial \lambda_{2}^{2}}-2\lambda_{1}\lambda_{2}\frac{\partial^{2}W}{\partial \lambda_{2}\lambda_{1}}\;\;-2\frac{\lambda_{1}\lambda_{2}\left(\lambda_{2}\frac{\partial W}{\partial \lambda_{1}}-\lambda_{1}\frac{\partial W}{\partial \lambda_{2}}\right)}{\lambda_{1}-\lambda_{2}}$$

It should be noted that these expressions are a consequence of the same coaxiality between stress and strain measures that permits (2). As such, they are also rigorously valid only under the assumption of material isotropy. A simple substitution of the components of the principal Cauchy stresses (i.e. $t_{i}=\lambda_{i}\partial W/\partial \lambda_{i}$) from (2) into (11) and (12) produces

(14) $$\alpha =\frac{\lambda_{1}^{2}}{\lambda_{1}^{2}-\lambda_{2}^{2}}\left(t_{1}-t_{2}\right)$$

(15) $$\gamma =\frac{\lambda_{2}^{2}}{\lambda_{1}^{2}-\lambda_{2}^{2}}\left(t_{1}-t_{2}\right)$$

While (14) and (15) are model-independent, it is clear that no similar substitutions can be made for *β* without producing derivatives of the components of ***t***. At this point, in order to make any further inferences about the wall stress, it is necessary to assume a constitutive model. The Demiray-Fung constitutive model [33], [34] happens to be a particularly strategic choice for the purpose of producing an expression for *β* of a similar form as that of (14) and (15). Its strain energy function can be expressed in terms of the first invariant of **B** (i.e. $I_{1}=tr(\text{B})=\lambda_{1}^{2}+\lambda_{2}^{2}+\lambda_{3}^{2}$) as follows.


(16) $$W=\frac{\mu_{0}}{2b}\left(e^{b\left(I_{1}-3\right)}-1\right)$$

Where *μ*_0_ is the initial shear modulus of the medium and *b* is a unitless parameter that determines the rate of strain stiffening within the medium. Substituting (16) into (13) produces the following expression for *β* for this model

(17) $$2\beta =2\left(\lambda_{1}^{4}+\lambda_{2}^{4}-2\lambda_{1}^{2}\lambda_{2}^{2}\right)\mu_{0}b\left(e^{b\left(I_{1}-3\right)}\right)\;\;+2\left(\lambda_{1}^{2}+\lambda_{2}^{2}\right)\mu_{0}\left(e^{b\left(I_{1}-3\right)}\right)$$

Likewise, an expression for stresses can be produced by substituting (16) into (2) and subtracting the components to eliminate *p*, which produces

(18) $$t_{1}-t_{2}=\lambda_{1}\frac{\partial W}{\partial \lambda_{1}}-\lambda_{2}\frac{\partial W}{\partial \lambda_{2}}=\left(\lambda_{1}^{2}-\lambda_{2}^{2}\right)\frac{\partial W}{\partial I_{1}}\;\;=\left(\lambda_{1}^{2}-\lambda_{2}^{2}\right)\mu_{0}e^{b\left(I_{1}-3\right)}$$

Thus, an expression for *β* in terms of stress can be produced by substituting (18) into (17), which results in

(19) $$2\beta =\frac{\left(\lambda_{1}^{4}+\lambda_{2}^{4}-2\lambda_{1}^{2}\lambda_{2}^{2}\right)b+\lambda_{1}^{2}+\lambda_{2}^{2}}{\lambda_{1}^{2}-\lambda_{2}^{2}}\left(t_{1}-t_{2}\right)$$

Now the only material parameter remaining is the nonlinearity parameter, *b*, which either must be assumed or estimated.

Having *γ*, *α* and *β* in terms of stress and deformation now permits (9) to be defined in terms of pressure and geometric measures. For simplicity, we collect the principal stretch terms under the following coefficients, *A* and *B*, as follows.


(20) $$A=\frac{\lambda_{1}^{2}}{\lambda_{1}^{2}-\lambda_{2}^{2}}=\frac{{\left(\frac{V}{V_{0}}\right)}^{2/3}}{{\left(\frac{V}{V_{0}}\right)}^{2/3}-{\left(\frac{V_{0}}{V}\right)}^{4/3}}$$

(21) $$B=\frac{\left(\lambda_{1}^{4}-\lambda_{2}^{4}-2\lambda_{1}^{2}\lambda_{2}^{2}\right)b+\lambda_{1}^{2}+\lambda_{2}^{2}}{\lambda_{1}^{2}-\lambda_{2}^{2}}\;\;=\frac{\left({\left(\frac{V}{V_{0}}\right)}^{8/3}+{\left(\frac{V_{0}}{V}\right)}^{16/3}-{\left(\frac{V}{V_{0}}\right)}^{2/3}{\left(\frac{V_{0}}{V}\right)}^{4/3}\right)b}{{\left(\frac{V}{V_{0}}\right)}^{2/3}-{\left(\frac{V_{0}}{V}\right)}^{4/3}}\;\;+\frac{{\left(\frac{V}{V_{0}}\right)}^{2/3}+{\left(\frac{V_{0}}{V}\right)}^{4/3}}{{\left(\frac{V}{V_{0}}\right)}^{2/3}-{\left(\frac{V_{0}}{V}\right)}^{4/3}}$$

Propogating all substitutions from (14), (15) and (19) into (9) and substituting for *t*_1_ − *t*_2_ with (5) produces the dispersion equation shown in (22) at the bottom of the page. Note also that $h=\lambda_{2}h_{0}=h_{0}{\left(V_{0}/V\right)}^{2/3}$ and $r={\left(3/4V/\pi \right)}^{1/3}$. Thus (22) is fully defined in terms of pressure (*P*), solid and fluid wave densities and wave speeds (*ρ*, *ρ*^*F*^, *c*_*p*_ and *c*), and compartment geometric measurements (*V* and *h*_0_).

## III. Methods

### A. Ex Vivo Bladder Measurements

UBV measurements collected from *ex vivo* pig bladders previously reported in [26] were used to validate this analysis. 9 *ex vivo* bladders were measured before and after a cross-linking treatment with formalin. During measurements, the bladder was placed in a water tank at room temperature. The bladders had initial filling volumes ranging from 190 to 330 ml. A syringe was used to add water to the bladder at 10 ml increments up to a total of 280 ml of added fluid and a pressure gauge (Omegadyne, Inc., Sunbury, OH, USA) was used to measure the compartment pressure of the bladder. A programmable ultrasound system (Verasonics, Kirkland, WA, USA) equipped with a linear array ultrasound transducer (L7–4, Philips Healthcare, Andover, MA) was used to collect UBV measurements.

Lamb waves were excited at the wall of the bladder through acoustic radiation force by a focused ultrasound beam with 400 *μ*s tone burst. The aperture size of the beam was 64 elements (corresponding to approximately 20 mm) and the modulation frequency of the burst was 4 MHz. A voltage of up to 90 V was applied to the active elements during the tone burst. Ultra-fast ultrasound imaging was used to capture the resulting wave propagation using transmit-receive pulses with a 5 MHz modulation frequency. Planewave images were acquired at a pulse repetition frequency of 12,000 Hz with 3 planewave angles (−4°, 0°, 4°) for coherent compounding, resulting in 4000 frames per a second. The beamformed in-phase/quadrature (IQ) data from these imaging frames were exported from the Verasonics system for subsequent processing. The described UBV acquisition sequence was repeated 5 times at each filling volume with the acoustic radiation force pulse and imaging frames positioned identically for all 5 acquisitions.


(22) $$\frac{V_{0}^{2}}{V^{2}}A\left(P\frac{r}{4h}\right)\sqrt{\frac{\frac{\rho c^{2}}{P\frac{r}{4h}}-2B}{2\frac{V_{0}^{2}}{V^{2}}A}+\frac{\sqrt{{\left(2B\left(P\frac{r}{4h}\right)-\rho c^{2}\right)}^{2}-4\left(\frac{V_{0}^{2}}{V^{2}}A^{2}{\left(P\frac{r}{4h}\right)}^{2}-\frac{V_{0}^{2}}{V^{2}}A\left(P\frac{r}{4h}\right)\rho c^{2}\right))}}{2\frac{V_{0}^{2}}{V^{2}}A\left(P\frac{r}{4h}\right)}}\;\times {\left(1+\frac{\frac{\rho c^{2}}{P\frac{r}{4h}}-2B}{2\frac{V_{0}^{2}}{V^{2}}A}-\frac{\sqrt{{\left(2B\left(P\frac{r}{4h}\right)-\rho c^{2}\right)}^{2}-4\left(\frac{V_{0}^{2}}{V^{2}}A^{2}{\left(P\frac{r}{4h}\right)}^{2}-\frac{V_{0}^{2}}{V^{2}}A\left(P\frac{r}{4h}\right)\rho c^{2}\right))}}{2\frac{V_{0}^{2}}{V^{2}}A\left(P\frac{r}{4h}\right)}\right)}^{2}\;\times \tanh\left(\sqrt{\frac{\frac{\rho c^{2}}{P\frac{r}{4h}}-2B}{2\frac{V_{0}^{2}}{V^{2}}A}+\frac{\sqrt{{\left(2B\left(P\frac{r}{4h}\right)-\rho c^{2}\right)}^{2}-4\left(\frac{V_{0}^{2}}{V^{2}}A^{2}{\left(P\frac{r}{4h}\right)}^{2}-\frac{V_{0}^{2}}{V^{2}}A\left(P\frac{r}{4h}\right)\rho c^{2}\right))}}{2\frac{V_{0}^{2}}{V^{2}}A\left(P\frac{r}{4h}\right)}}kh\right)\;-\frac{V_{0}^{2}}{V^{2}}A\left(P\frac{r}{4h}\right)\sqrt{\frac{\frac{\rho c^{2}}{P\frac{r}{4h}}-2B}{2\frac{V_{0}^{2}}{V^{2}}A}-\frac{\sqrt{{\left(2B\left(P\frac{r}{4h}\right)-\rho c^{2}\right)}^{2}-4\left(\frac{V_{0}^{2}}{V^{2}}A^{2}{\left(P\frac{r}{4h}\right)}^{2}-\frac{V_{0}^{2}}{V^{2}}A\left(P\frac{r}{4h}\right)\rho c^{2}\right))}}{2\frac{V_{0}^{2}}{V^{2}}A\left(P\frac{r}{4h}\right)}}\;\times {\left(1+\frac{\frac{\rho c^{2}}{P\frac{r}{4h}}-2B}{2\frac{V_{0}^{2}}{V^{2}}A}+\frac{\sqrt{{\left(2B\left(P\frac{r}{4h}\right)-\rho c^{2}\right)}^{2}-4\left(\frac{V_{0}^{2}}{V^{2}}A^{2}{\left(P\frac{r}{4h}\right)}^{2}-\frac{V_{0}^{2}}{V^{2}}A\left(P\frac{r}{4h}\right)\rho c^{2}\right))}}{2\frac{V_{0}^{2}}{V^{2}}A\left(P\frac{r}{4h}\right)}\right)}^{2}\;\times \tanh\left(\sqrt{\frac{\frac{\rho c^{2}}{P\frac{r}{4h}}-2B}{2\frac{V_{0}^{2}}{V^{2}}A}-\frac{\sqrt{{\left(2B\left(P\frac{r}{4h}\right)-\rho c^{2}\right)}^{2}-4\left(\frac{V_{0}^{2}}{V^{2}}A^{2}{\left(P\frac{r}{4h}\right)}^{2}-\frac{V_{0}^{2}}{V^{2}}A\left(P\frac{r}{4h}\right)\rho c^{2}\right))}}{2\frac{V_{0}^{2}}{V^{2}}A\left(P\frac{r}{4h}\right)}}kh\right)\;+\frac{\rho^{F}c^{2}}{\xi }\left(2\frac{\sqrt{{\left(2B\left(P\frac{r}{4h}\right)-\rho c^{2}\right)}^{2}-4\left(\frac{V_{0}^{2}}{V_{1}^{2}}A^{2}{\left(P\frac{r}{4h}\right)}^{2}-\frac{V_{0}^{2}}{V^{2}}A\left(P\frac{r}{4h}\right)\rho c^{2}\right))}}{2\frac{V_{0}^{2}}{V^{2}}A\left(P\frac{r}{4h}\right)}\right)=0$$

Particle velocities were estimated from the acquired IQ frames using a phase-based autocorrelation technique [35]. A median filter with a 3 × 3 pixel window was then applied to the frames of particle velocity data. After adjusting for the curvature of the bladder wall [15], the axial particle velocities were averaged by taking the median particle velocity along the thickness of the bladder wall. The frequency-resolved wave speed was then estimated as in [36] by first applying a 2D Fourier transform and collecting the peaks from the 2D magnitude spectrum. The phase velocity can then be estimated from the values of *k* and *f* associated with each peak via *c* = 2*πf/k*. For each frequency, the median phase velocity over 5 acquisitions was then collected and then used for subsequent curve fitting.

Median-based averaging and filtering was used throughout the processing due to the nonlinear nature of the particle velocity estimation and k-space wave speed estimation. Noisy inputs to such nonlinear functions produce outlier estimates that tended to dominate the estimate when a mean was used for averaging. For the current experiments, noisy inputs were a consequence of the lack of acoustic signal away from the bladder wall and the occasional air bubble (See Appendix A for a detailed explanation). Use of median-based averaging and filtering prevented outlier measurements from severely contaminating the final estimation.

In order to characterize the deviation in the phase velocity for each bladder, we report the variance in the phase velocity at a given frequency and fill volume. Because variances are additive, these variances can be averaged across fill volumes and frequencies to give a single average variance that characterizes the amount of noise in the measurements collected in individual bladder experiments. We hereafter refer to this average as the mean phase velocity variance (MPVV).

### B. Pressure Estimation

Estimation of *P* was accomplished by optimization-based curve fitting applied to (22) (See Fig. 1(d) for example curve fit). Fitting was applied using Matlab’s optimization toolbox (MATLAB 2019a, Mathworks Inc, Natick, MA, USA) using a simplex search method to find a minimum [37]. If ${\text{Θ}}_{i}(P)$ is the value of the left side of (22) for the *i*th frequency, then *P* is estimated by

(23) $$P^{\left(est\right)}=\text{argmin}\left\{\sum_{i=1}^{N}{\text{Θ}}_{i}(P)*\bar{{\text{Θ}}_{i}(P)}\right\}$$

The bar above $\text{Θ}{\left(P\right)}_{i}$ denotes its complex conjugate, which is used to ensure a real-valued quantity. The initial guess for minimization for *P* was set to 100 mmHg. The frequency range used for fitting was 150–500 Hz. This range was selected in order to match the range used in in vivo UBV measurements [16].

The nonlinearity parameter *b* was set to an assumed value of 5. This is the same value used in [25] for modeling vascular tissue and is comparable to the measurement of fibroglandular tissue and *ex vivo* liver tissue [20]. As such, it is a reasonable assumed value for soft tissue, albeit not specialized to bladder tissue in particular. The undeformed volume *V*_0_ was inferred using (8) and assuming an undeformed thickness of 5.5 mm (i.e. *h*_0_ = 2.75 mm), which is in line with the average bladder wall thicknesses reported in [38] and [39]. Since an estimate of *V*_0_ can be inferred from each measured fill volume and thickness, fitting was applied for each experiment using the average of the inferred *V*_0_. Since *b* and *h*_0_ are fixed parameters, an evaluation of the sensitivity in the overall pressure estimation to these parameters are included as an appendix (See Appendix B).

### C. Performance Measures and Statistical Analysis

For descriptive statistics, we considered root mean squared deviation (RMSD), mean error (ME) and relative mean error (RME). These quantities are defined respectively as

(24) $$RMSD=\sqrt{1/N\sum i=1^{N}{\left(P_{i}^{\left(est\right)}-P_{i}^{\left(meas\right)}\right)}^{2}}$$

(25) $$ME=1/N\sum_{i=1}^{N}\left(P_{i}^{\left(est\right)}-P_{i}^{\left(meas\right)}\right)$$

(26) $$RME=100\times \left(1/N\sum_{i=1}^{N}\left(\frac{(P_{i}^{\left(est\right)}-P_{i}^{\left(meas\right)}}{P_{i}^{\left(meas\right)}}\right)\right)$$

where the added superscripts denote estimated (est) versus measured (meas) pressures for the *i*th volume and *N* is the number of estimates.

The degree of agreement between *P*^(*est*)^ and *P*^(*meas*)^ was assessed using concordance analysis. In particular, Lin’s concordance correlation coefficient (CCC) was used to characterize the statistical degree of agreement between *P*^(*est*)^ and *P*^(*meas*)^. Analogous to a classic correlation coefficient, Lin’s CCC takes on a value between −1 and 1, where 1 corresponds to perfect statistical agreement and 0 corresponds to no statistical agreement. However Lin’s CCC includes deviation away from ideal one-to-one agreement and thus characterizes both precision and accuracy. Lin’s CCC and its associated confidence intervals were calculated using the mixed effects model approach for repeated measures proposed in [40]. This repeated measures model was adopted in order to avoid artificially lowering confidence intervals due to having multiple measurements collected from independent bladders. Using this approach, Lin’s CCC is calculated from the variance components of the mixed effects model as follows:

(27) $$\frac{\sigma_{s}^{2}+\sigma_{sn}^{2}}{\sigma_{s}^{2}+\sigma_{sn}^{2}+\sigma_{sm}^{2}+\sigma_{mn}^{2}+\sigma_{e}^{2}}$$

Here *σ*^2^ denotes the given variance component and the subscripts denote the effect or interaction of the given measurement’s bladder(*s*), measurement number (*n*), and measurement method (*m*). Measurements were imported into R [41] and the ‘ccclon’ function from the ‘cccrm’ package [42] was used to estimate Lin’s CCC and its 95% confidence interval.

## IV. Results

**Fig. 2.**
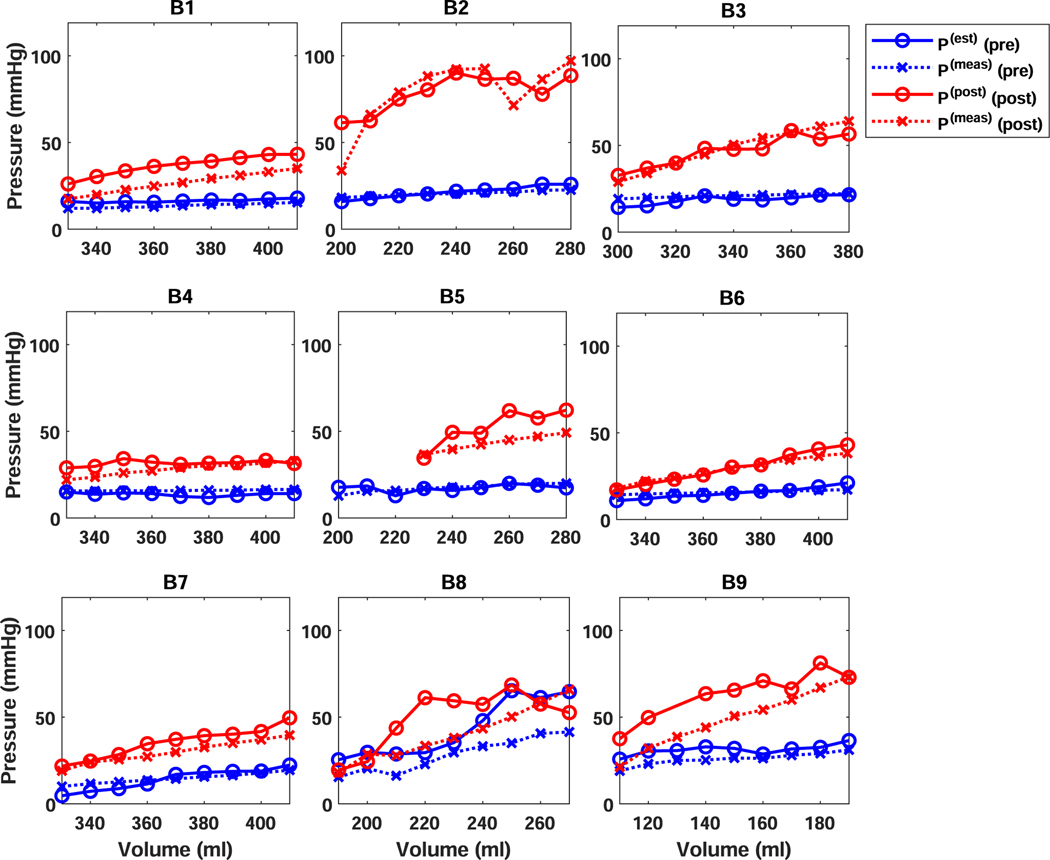
Measured (dotted line; x markers) and UBV estimated (dashed line; circular markers) pressure as a function of volume for all 8 bladders. Missing values correspond to volumes were all acquisitions had an SNR below 2. (Note: B1=Bladder 1) The missing values in Bladder 5 and Bladder 9 were measurements where no wave signal was produced due to an acquisition error at the time of the experiment.

Fig. 2 shows vessel pressures estimated from the proposed analysis plotted along with the vessel pressure measured by the pressure gauge as a function of bladder volume. These plots illustrate how the analysis performs at recreating the underlying pressure from wave propagation and deformation (i.e. volume) measurements.

Table I reports the peformance measures (i.e. RMSD, ME and RME), *V*_0_ value used in the pressure estimation, and the MPVV values for pressures for the experiments shown in Fig. 2. These results show that the estimated pressures were broadly in agreement with the measured pressure (RMSD: 8.17 (mmHg); ME: 3.40 (mmHg); RME: 11.48 (%).

**Fig. 3.**
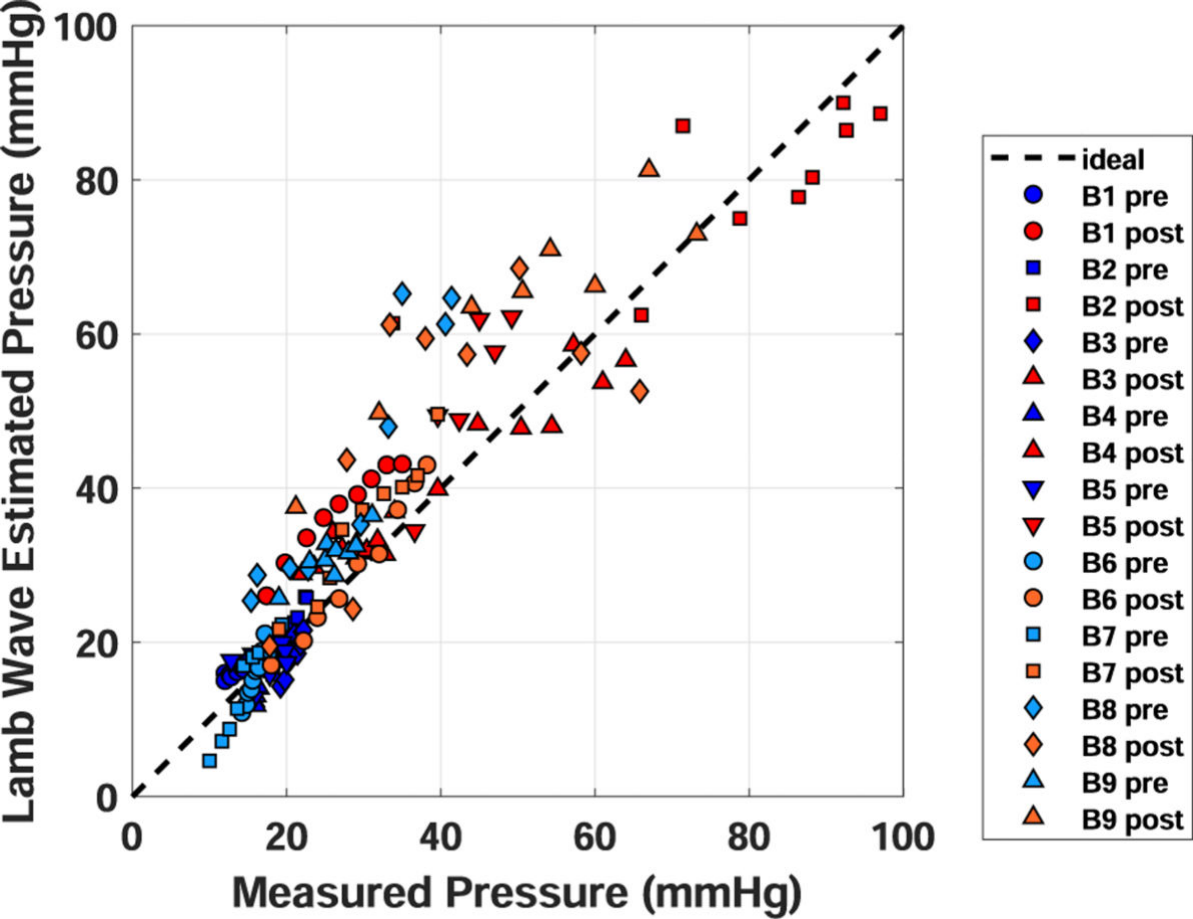
Combined scatter plot of Lamb wave inferred pressure versus measured pressure from all bladders before and after formalin.

Fig. 3 displays a scatter plot of all measurements from the 9 bladders. It can be seen that the combined data appear to align with an ideal one-to-one match between *P*^(*est*)^ and *P*^(*meas*)^ (i.e. the line corresponding to ideal concordance). The CCC calculated from the concordance analysis was 0.82 with a 95% confidence interval of 0.66 − 0.91.

Although the combined estimates were in broad agreement with the estimated pressures, there was variability in the errors observed for individual bladder experiments (1.57–16.55 (mmHg); ME: −2.31–14.55 (mmHg); RME: −14.46–51.55 (%)) and a few bladder experiments displayed substantial errors. We noticed that most of those bladder experiments with substantial differences between *P*^(*est*)^ and *P*^(*meas*)^ also showed large MPVVs. In particular, bladder 8 before and after cross-linking and bladder 9 after cross-linking showed substantial errors. As a post-hoc analysis, we considered the effect of excluding bladder experiments that had MPVVs that were above the combined average of 18.79 *m*^2^*s*^−2^. We found that the concordance increased to 0.90 (0.78–0.95) and that the combined performance measures were also improved (See last row in Table I).

## V. Discussion

In this study, we proposed and derived a mechanical analysis that directly relates the pressure in a spherical pressure-vessel to measurements of the dispersion curve of a Lamb wave. We used the analysis to estimate pressure from UBV measurements collected from *ex vivo* pig bladders at varied filling volumes and elasticities. The resulting pressure estimates were found to be in broad agreement with measured pressure (Figs. 2 and 3).

The apparent increases in estimation errors at large pressures are likely explained by the increased errors in wave speed estimates expected at higher wave speeds. To see why this is the case, one can consider how frequency dependent wave speed is estimated from the 2D-FT magnitude spectrum (Fig. 1(c)). Since the wave speed is derived from the estimated wave number at a given frequency as *c* = 2*πf/k*, a larger value for *c* corresponds to a smaller value for *k*. As such, in the case where the expected error in the estimates of *k* are constant, larger wave speeds are expected to correspond to a larger error in estimated wave speed.

The proposed mechanistic analysis offers two major advantages over simple empirical association or curve fitting for the purpose of pressure estimation in biological compartments. The first advantage is that the model can be expected to generalize to compartments of varied characteristics (e.g. elasticity or size) so long as said characteristics are contained within the analysis. This is demonstrated here by the general agreement between the measured and estimated pressures shown in Fig. 3.

The second advantage a mechanistic analysis offers is a framework for refinement toward more accurate estimation. Because the correlation between pressure and wave speed are determined mechanistically, greater accuracy can, in principle, be attained by examining the simplifying assumptions in the analysis and adapting the analysis and measurements to incorporate additional factors. Furthermore, evaluating the error due to neglecting such factors is feasible *in situ* because the analysis relates two quantities that are directly measurable for many pressurized compartments (i.e. Lamb wave speed and internal pressure). With this in mind, we wish to recognize that experimental verification remains warranted when applying or adapting the proposed approach to novel conditions that are beyond what was explored here.

We now discuss some of the mechanical factors that are neglected in this analysis as well as their potential significance to the mechanics of these current experiments and relevance to adaptations to other biological compartments. Although assuming a spherical compartment is in line with similar biomechanical analysis of the bladder [23], [24] it is also recognized that the geometry of the bladder deviates from this ideal case [43], [44]. Non-spherical geometries will alter the relation between wall-stress and internal pressure [32].

The significance of wall-thickness is another factor that deserves discussion. As we have shown in Section II-B, the assumption of a thin wall is tied to the assumption of a homogenous stress across the compartment wall that is needed for the dispersion analysis. Although the stress analysis for a thick-walled vessel is well-known [23], [24], the wave analysis for a non-homogenous stress is substantially more complicated than what is proposed here. Such an analysis would likely require a numerical approach such as that in [45] in order to determine the dispersion curves for a given pressure and set of material properties. It is worthy of future consideration, as it would be insightful for determining the error in this current approach that is associated with the thin-wall assumption, which would be relevant for its generalization to other biological compartments.

The dynamic contractile behavior of the *in vivo* bladder is a relevant constative factor that is not explicitly handled in this current analysis. Such contractions can produce dynamic variation in pressure as is the case during filling where detrusor overactivity is present or during the onset of voiding in healthy bladders. Although further investigation is needed to assess the influence of active contraction on the proposed pressure estimation, there is reason to hypothesis that the such estimations would remain valid. To the degree that tissue contractile behavior can be modeled as a dynamic change in the tissue’s linear elasticity, the effect of contraction would be to produce fluctuations in either or both the stretch and stress of the tissue. Assuming such fluctuations are on a large time scale relative to the Lamb wave propagation and have a negligible influence on the strain stiffening parameter, b, their effect would be to shift the values of λ_1_, λ_2_ and (*t*_1_ − *t*_2_) in (14), (15) and (19). This would result in the expected fluctuations in pressure due to contraction as well as fluctuations in wave speed. Furthermore, a recent work on skeletal muscle [46] demonstrated that the effect of active contraction on shear wave speed could be effectively modeled using acoustoelastic analysis with no additional constative assumptions related to active contraction. Therefore, although the effect of active contractile behavior is a factor worth further consideration, there are mechanistic and empirical reasons to hypothesis that it is not likely to confound the proposed pressure estimation.

The proposed approach also assumes material isotropy and a static fluid. While these are reasonable assumptions in the case of bladder, they are likely to be substantial limitations to the application of the approach to other biological compartments. For instance, anisotropy and substantial fluid flow at the boundary of the compartment wall are to be anticipated for the ventricular wall of the heart and for blood vessels. Likewise, while fluid flow might not be as substantial a consideration for the diaphragm, anisotropy would remain a potentially substantial confounder to the analysis. Refinements of the approach to incorporate such characteristics are by no means trivial. In the case of anisotropy, while a similar Lamb wave analysis has been recently proposed [47], the coaxiality between stress and strain measures that permit this approach would no longer hold in the case of material anisotropy. Likewise, the presence of significant fluid flow would need to be incorporated into the fluid mechanics governing the wave propagation, such as was done in [48]. In such scenarios, the proposed approach may still offer a useful first approximation for studying the mechanics of these compartments, though greater estimation errors would be expected.

Considerations of dynamic pressure loading and tissue viscoelasticity are also neglected by the proposed analysis. This is both a theoretical and experimental consideration that would require refinements to both in order to investigate. Experimentally, investigating the effect of dynamic loading would require time-resolved pressure measurements that are precisely synchronized with the UBV acquisitions. This would permit the assessment of the stress relaxation expected for a finitely deformed viscohyperelastic solid [24], [49]. Such relaxation effects would also need to be incorporated into the wave model, as well as the viscoelastic dispersion and attenuation expected for the wave propagation [29], [36]. Such a refinement is not trivial, as it would need to start at the equations of motion used to derive the dispersion equation.

A final pragmatic consideration is in the feasibility of translating the proposed analysis to *in vivo* pressure estimations in the bladder. Although some of the sources of variation in these current experiments are not likely to be as substantial in the in vivo case (e.g. air bubbles; see Appendix I), the complexity of the imaging scenario is increased and the targeted wave signal is weaker than in these current experiments due to the added acoustic attenuation and depth during imaging. The development of a robust wave tracking approach that will allow for translation of this approach to in vivo bladder measurements is a topic that we are actively investigating.

## VI. Conclusion

In this study, we proposed a mechanical analysis for estimating pressure from ultrasonically measured lamb wave speed in a pressurized biological compartment. Our analysis coupled acoustoelastic analysis of Lamb waves with spherical pressure-vessel analysis. We demonstrated that the analysis could be used to infer bladder pressure in *ex vivo* pig bladders with varied filling volumes and elasticities (i.e. formalin cross-linking). The general approach has potential applications as a means of noninvasive pressure estimation in biological compartments which is relevant to a range of biological structures.

## Figures and Tables

**Fig. 4.**
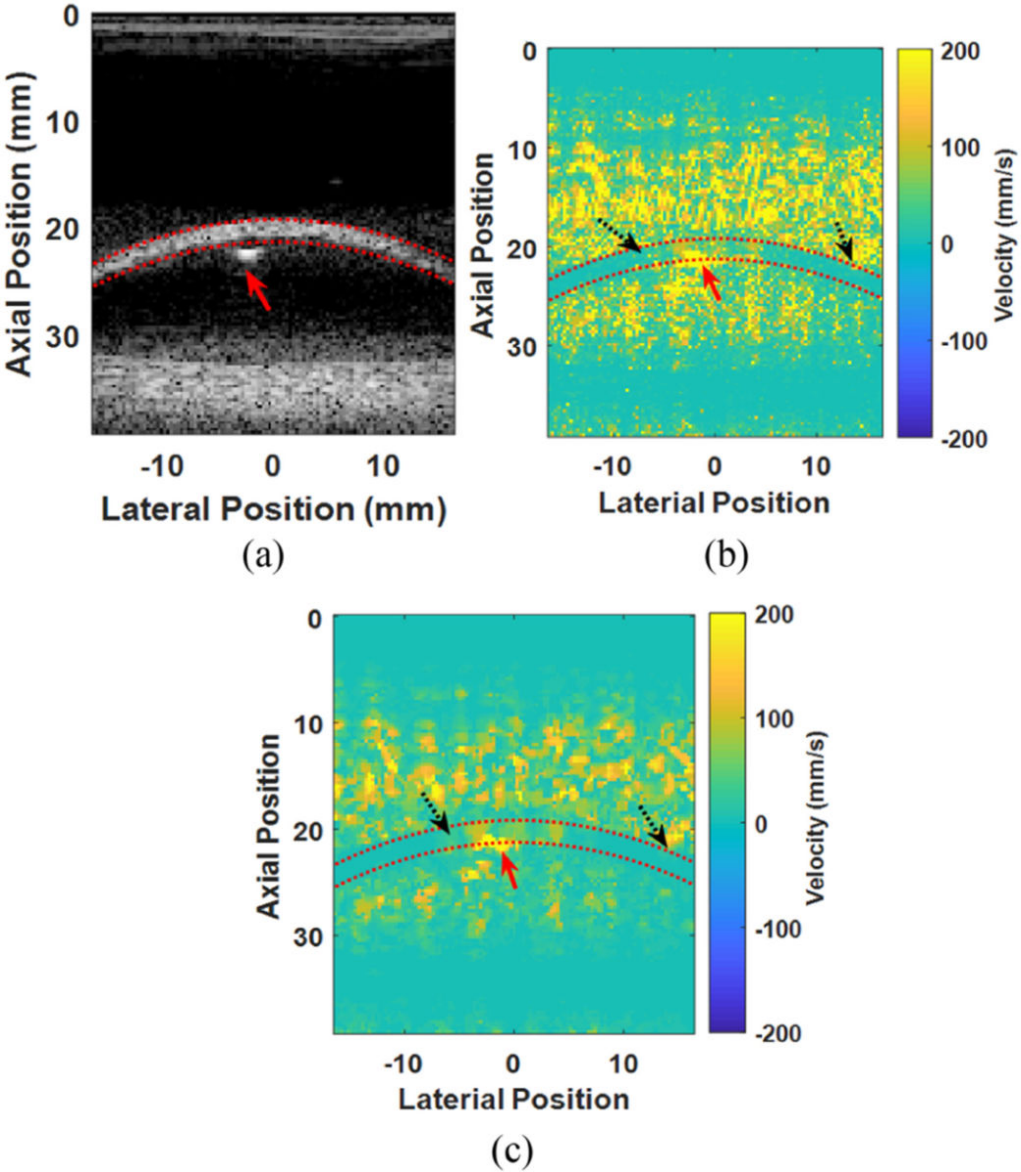
Example b-mode (a) and particle velocity images collected after the pushing pulse without (b) and with (c) application of a median filter. The red-dashed lines outline the bladder wall. A single air bubble can be seen in the b-mode image (red arrow) outside of the bladder wall. Accompanied artifacts in the particle velocity are observabled above the bubble (red solid arrow). Outlier velocity pixels in the bladder wall and at the edge of the bladder wall (black dotted arrows) can be observed in the raw particle velocity image (b). These outlier velocity pixels are removed after applying the median filter (c), though the bubble artifact remains.

**TABLE I:** Performance Measures, Reference Volumes and MPVV for the 9 *Ex Vivo* Bladders Pre and Post Formalin Treatment. Measures for Individual Bladder Experiments are Shown as Well as Combined Measure for All Bladder Experiements (Combined) and for Bladder Experiments with MPVV Values Below 18.79 *m*^2^*s*^−2^ (MPVV<18.79). (Note: RMSD = Root Mean Squared Deviation; ME=mean Error; RME=relative Mean Error; MPVV = Mean Phase Velocity Variance and B1= Bladder 1)

	RMSD (mmHg)	ME (mmHg)	RME (%)	*V*_0_ (ml)	MPVV (*m*^2^*s*^−2^)
Bl (pre)	2.77	2.72	20.51	65.44	0.70
Bl (post)	9.96	9.89	39.14	61.60	0.03
B2 (pre)	1.57	0.72	3.04	47.59	12.74
B2 (post)	12.16	−0.55	5.35	53.87	6.99
B3 (pre)	2.69	−2.35	−11.47	51.78	42.76
B3 (post)	5.09	−1.69	−1.31	57.31	0.59
B4 (pre)	2.50	−2.31	−14.46	56.22	4.36
B4 (post)	4.54	3.41	13.52	44.03	17.04
B5 (pre)	2.30	−0.09	1.07	25.68	12.15
B5 (post)	10.61	8.70	19.13	38.02	17.88
B6 (pre)	2.25	−0.30	−2.72	45.8	4.7
B6 (post)	2.17	0.67	1.09	49.44	0.58
B7 (pre)	3.08	−0.60	−8.103	47.24	57.57
B7 (post)	5.75	5.18	16.61	53.29	7.42
B8 (pre)	16.55	14.55	51.55	40.16	84.64
B8 (post)	15.15	8.55	26.43	31.60	27.63
B9 (pre)	5.14	4.73	19.22	41.79	0.33
B9 (post)	14.74	12.76	33.03	28.05	40.17

Combined	8.17	3.40	11.48	-	18.79
MPW< 18.79	5.87	2.02	7.73	-	6.58

**Fig. 5.**
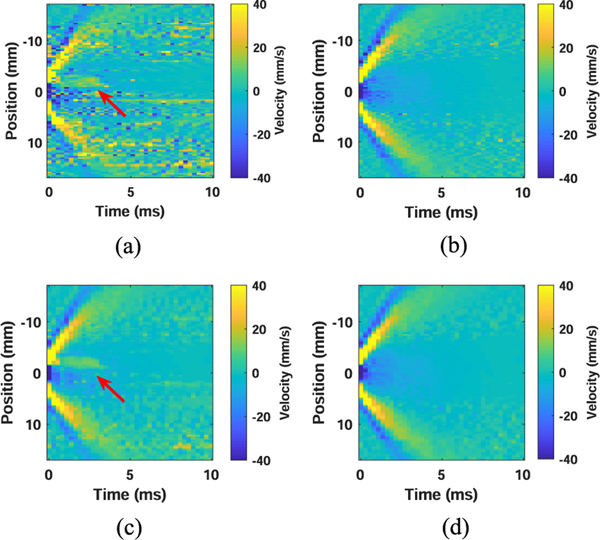
Averaged particle velocities across the thickness of the bladder walll from the example shown in Fig. 4 with varied filtering and averaging methods. Averaged particle velocities are shown with (c, d) and without (a, c) applying the median filtering shown in Fig. 4(c) prior to averaging. Mean based averaging is used in (a, c) and median based averaging is used in (b, d). An artifact in the approximate location of the air bubble observed in Fig. 4 can be seen when using mean based averaging (red arrow).

**Fig. 6.**
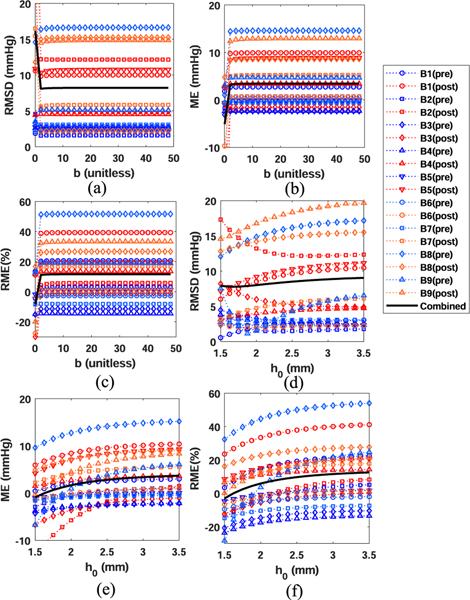
Effect of varying the strain stiffening parameter, *b*, (a, b, c) and the assumed wall thickness (d, e, f) on the RMSD, ME and RME of the estimation. Note that the thickness is 2*h*_0_.

## Appendix A Median-Based Averaging for Processing

As described in Section III-A, median-based filtering and averaging was used to derive the final phase velocity for estimation. The following example illustrates the motivation for this median-based approach in relation to the nonlinear nature of the processing steps and several imaging artifacts produced that are a consequence of the experimental setup. We can see in Fig. 4(a), the b-mode of the acquisition shows a well-defined, hyperechoic bladder wall surrounded by the anechoic water of the water bath. A single air bubble can be seen near the bladder wall. The raw particle velocity image (Fig. 4(b)), here shown at the first time sample, shows very large variation around the bladder wall. This variation is a consequence of the lack of an acoustic signal in the anechoic water, as the velocity estimate is derived from the phase of electric noise. Such noisy pixels can sometime be included in the axial averaging along the bladder wall. Within the bladder wall, we can also see some large outlier pixels as well as an artifact that appears to be due to the air bubble observed in the b-mode. The latter are removed through applying a median filter to the particle velocity images (Fig. 4(c) and Fig. 5(c)). The artifact from the air bubble can be removed by median averaging along the axial wall (Fig. 5(d)) in place of mean averaging (Fig. 5(c)).

## Appendix B Sensitivity of Estimation to Change in Fixed Parameters

Because *b* and *h*_0_ are fixed parameters in the estimation, their effect on the pressure estimation was assessed by varying the individual parameters over a wide range. For b, its range was varied across 3 orders of magnitude. For the half thickness, a range of 1.5 mm to 3.5 mm was considered. This range encompasses 2 standard deviations from the mean reported in [39] as well as the range reported in [38]. Fig. 6 shows the change in RMSD, ME and RME for individual bladders and combined bladders when varying these parameters. From Fig. 6(a-c) it can be seen that the estimation is mostly unaffected by variation in the strain stiffening parameter except for values of the parameter near 0. This would seem to indicate that, while a degree of strain stiffening is required for an accurate estimation, the estimation is quite insensitive to the precise value of b. In the case of the half thickness, Fig. 6(d(a-f)) shows more substantial variation in the estimates for individual bladders, especially for thinner wall thicknesses. However, the RMSD of the overall estimation changed by only 1.11 mmHg (from 7.93 mmHg to 9.04 mmHg) across the range of *h*_0_ values.

## References

[1] Casha AR , “A mathematical model for pressure-based organs behaving as biological pressure vessels,” J. Theor. Biol, vol. 450, pp. 37–42, 2018. [Online]. Available: http://www.sciencedirect.com/science/article/pii/S0022519318302170 29705490 10.1016/j.jtbi.2018.04.034

[2] Schepens T, Fard S, and Goligher EC, “Assessing diaphragmatic function,” Respir. Care, vol. 65, no. 6, pp. 807–819, 2020. [Online]. Available: http://rc.rcjournal.com/content/65/6/807.abstract 32457172 10.4187/respcare.07410

[3] Xu G, Li F, and Mao Y, “Portal pressure monitoring-state-of-the-art and future perspective,” Ann. Transl. Med, vol. 7, no. 20, pp. 583–583, 2019. [Online]. Available: https://pubmed.ncbi.nlm.nih.gov/31807564 31807564 10.21037/atm.2019.09.22 PMC6861775

[4] Rick AN and Blase AC, “Hemodynamics in the cardiac catheterization laboratory of the 21st century,” Circulation, vol. 125, no. 17, pp. 2138–2150, 2012, doi: 10.1161/CIRCULATIONAHA.111.060319. 22547754

[5] Mousa RTA, Dossary NA, and Hashim H, “The role of urodynamics in females with lower urinary tract symptoms,”ArabJ.Urol,vol.17,no.1, pp. 2–9, 2019. 10.1080/2090598X.2019.1589931 PMC6583751 31258939

[6] Peyronnet B. , “A comprehensive review of overactive bladder pathophysiology: On the way to tailored treatment,” Eur. Urol, vol. 75, no. 6, pp. 988–1000, 2019. 30922690 10.1016/j.eururo.2019.02.038

[7] Bodnar Z, “Polycompartment syndrome - Intra-abdominal pressure measurement,”Anaesthesiol.IntensiveTher,vol.51,no.4,pp. 316–322,2019, doi: 10.5114/ait.2019.87474. 31517472

[8] Liao J-Y , “Monitoring bladder compliance using end filling detrusor pressure: Clinical results and related factors,” Taiwanese J. Obstet. Gynecol, vol. 54, no. 6, pp. 709–715, 2015. [Online]. Available: https://www.sciencedirect.com/science/article/pii/S1028455915002338 26700990 10.1016/j.tjog.2015.10.003

[9] Farag FF and Heesakkers JP, “Non-invasive techniques in the diagnosis of bladder storage disorders,” Neurourol. Urodynamics, vol. 30, no. 8, pp. 1422–1428, 2011. [Online]. Available: https://onlinelibrary.wiley.com/doi/abs/10.1002/nau.21155 10.1002/nau.21155 21780168

[10] Andersen O. , “Estimating left ventricular filling pressure by echocardiography,” J. Amer. College Cardiol, vol. 69, no. 15, pp. 1937–1948, 2017. [Online]. Available: https://browzine.com/articles/91017599 10.1016/j.jacc.2017.01.058 28408024

[11] Tayebi S. , “A concise overview of non-invasive intra-abdominal pressure measurement techniques: From bench to bedside,” J. Clin. Monit. Comput, pp. 51–70, 2020. [Online]. Available: 10.1007/s10877-020-00561-4 32700152 PMC7889558

[12] Barr RG and Zhang Z, “Effects of precompression on elasticity imaging of the breast,” J. Ultrasound Med, vol. 31, no. 6, pp. 895–902, 2012. 22644686 10.7863/jum.2012.31.6.895

[13] Hug F, Tucker K, Gennisson J-L, Tanter M, and Nordez A, “Elastography for muscle biomechanics: Toward the estimation of individual muscle force,” Exercise Sport Sci. Rev, vol. 43, no. 3, pp. 125–133, 2015. 10.1249/JES.0000000000000049 25906424

[14] Martin JA , “Gauging force by tapping tendons,” Nature Commun, vol. 9, no. 1, pp. 1592–1601, 2018. [Online]. Available: 10.1038/s41467-018-03797-6 29686281 PMC5913259

[15] Nenadic I. , “Noninvasive evaluation of bladder wall mechanical properties as a function of filling volume: Potential application in bladder compliance assessment,” Plos One, vol. 11, no. 6, pp. 1–14, 2016. 10.1371/journal.pone.0157818 PMC4920425 27341340

[16] Bayat M. , “Correlation of ultrasound bladder vibrometry assessment of bladder compliance with urodynamic study results, ”PLOS ONE,vol.12, no. 6, 2017, Art. no. e0179598, doi: 10.1371/journal.pone.0179598. PMC5473568 28622388

[17] Bachasson D. , “Diaphragm shear modulus reflects transdiaphragmatic pressure during isovolumetric inspiratory efforts and ventilation against inspiratory loading,” J. Appl. Physiol, vol. 126, no. 3, pp. 699–707, 2019. [Online]. Available: https://journals.physiology.org/doi/abs/10.1152/japplphysiol.01060.2018 30730816 10.1152/japplphysiol.01060.2018

[18] Vejdani-Jahromi M. , “Measuring intraventricular pressure using ultrasound elastography,” J. Ultrasound Med, vol. 38, no. 5, pp. 1167–1177, 2019. [Online]. Available: https://onlinelibrary.wiley.com/doi/abs/10.1002/jum.14795 30218456 10.1002/jum.14795

[19] Wang Y. , “Bidirectional ultrasound elastographic imaging frame work for non-invasive assessment of the non-linear behaviour of a physiologically pressurized artery,” Ultrasound Med. Biol, vol. 45, no. 5, pp. 1184–1196, 2019. [Online]. Available: http://www.sciencedirect.com/science/article/pii/S0301562919300237 30876671 10.1016/j.ultrasmedbio.2019.01.014

[20] Jiang Y. , “Characterization of the nonlinear elastic properties of soft tissues using the supersonic shear imaging (SSI) technique: Inverse method, ex vivo and in vivo experiments,”Med.ImageAnal,vol.20,no.1, pp. 97–111, 2015b. 10.1016/j.media.2014.10.010 25476413

[21] Li G-Y and Cao Y, “Mechanics of ultrasound elastography,” in Proc. Math., Phys., Eng. Sci, vol. 473, no. 2199, 2017, Art. no. 20160841. [Online]. Available: https://pubmed.ncbi.nlm.nih.gov/28413350 10.1098/rspa.2016.0841 PMC5378248 28413350

[22] Nenadic IZ , “Ultrasound bladder vibrometry method for measuring viscoelasticity of the bladder wall,” Phys. Med. Biol, vol. 58, no. 8, pp. 2675–2695, 2013, doi: 10.1088/0031-9155/58/8/2675. 23552842

[23] Mangan R. and Destrade M, “Gent models for the inflation of spherical balloons,” Int. J. Non-Linear Mechanics, vol. 68, pp. 52–58, 2015.

[24] Pascalis RD , “The inflation of viscoelastic balloons and hollow viscera,” in Proc. Roy. Soc. A: Math., Phys. Eng. Sci, vol. 474, no. 2218, 2018, Art. no. 20180102. [Online]. Available: 10.1098/rspa.2018.0102

[25] Li G-Y , “Guided waves in pre-stressed hyperelastic plates and tubes: Application to the ultrasound elastography of thin-walled soft materials,” J. Mechanics Phys. Solids, vol. 102, pp. 67–79, 2017. [Online]. Available: http://www.sciencedirect.com/science/article/pii/S0022509616308250

[26] Bayat M. , “Acoustoelasticity analysis of transient waves for non-invasive in vivo assessment of urinary bladder,” Sci. Rep, vol. 9, no. 1, pp. 2441–2450, 2019, doi: 10.1038/s41598-018-38445-y. 30792448 PMC6385274

[27] Fovargue D. , “Towards noninvasive estimation of tumour pressure by utilising MR elastography and nonlinear biomechanical models: A simulation and phantom study,” Sci. Rep, vol. 10, no. 1, pp. 5588–5601, 2020, doi: 10.1038/s41598-020-62367-3. 32221324 PMC7101441

[28] Schaafs LA , “Quantification of aortic stiffness by ultrasound time-harmonic elastography: The effect of intravascular pressure on elasticity measures in a porcine model,” Invest. Radiol, vol. 55, no. 3, pp. 174–180, 2020. 31895220 10.1097/RLI.0000000000000618

[29] Nenadic IZ , “Phase velocities and attenuations of shear, lamb, and Rayleigh waves in plate-like tissues submerged in a fluid (L),” J. Acoustical Soc. Amer, vol. 130, no. 6, pp. 3549–3552, 2011. [Online]. Available: https://pubmed.ncbi.nlm.nih.gov/22225009 10.1121/1.3654029 PMC3253592 22225009

[30] Ogden RW, “Incremental statics and dynamics of pre-stressed elastic materials,” in Waves in Nonlinear Pre-Stressed Materials. Berlin, Germany: Springer, 2007, pp. 1–27.

[31] Ogden RW, Non-Linear Elastic Deformations. New York, NY, USA: Dover Publications, Inc., 1997, pp. 247–336.

[32] Yin FC, “Ventricular wall stress,” Circulation Res, vol. 49, no. 4, pp. 829–842, 1981. [Online]. Available: https://www.ahajournals.org/doi/abs/10.1161/01.RES.49.4.829 7023741 10.1161/01.res.49.4.829

[33] Demiray H, “A note on the elasticity of soft biological tissues,” J. Biomech, vol. 5, pp. 309–311, 1972. 4666535 10.1016/0021-9290(72)90047-4

[34] Fung YC, “Elasticity of soft tissues in simple elongation,” Amer. J. Physiol.-Legacy Content, vol. 213, no. 6, pp. 1532–1544, 1967. 10.1152/ajplegacy.1967.213.6.1532 6075755

[35] Loupas T, Powers J, and Gill R, “An axial velocity estimator for ultrasound blood-flow imaging, based on a full evaluation of the Doppler equation by mean of a 2-dimensional autocorrelation approach,” IEEE Trans. Ultrasonics Ferroelect. Freq. Control, vol. 42, no. 4, pp. 672–688, Jul. 1995.

[36] Nenadic IZ , “Lamb wave dispersion ultrasound vibrometry (LDUV) method for quantifying mechanical properties of viscoelastic solids,” Phys. Med. Biol, vol. 56, no. 7, pp. 2245–2264, 2011. [Online]. Available: https://pubmed.ncbi.nlm.nih.gov/21403186 21403186 10.1088/0031-9155/56/7/021 PMC3086697

[37] Lagarias JC , “Convergence properties of the Nelder-Mead simplex method in low dimensions,” SIAM J. Optim, vol. 9, no. 1, pp. 112–147, 1998.

[38] Morales-Orcajo E, Siebert T, and Böl M, “Location-dependent correlation between tissue structure and the mechanical behaviour of the urinary bladder,” Acta Biomaterialia, vol. 75, pp. 263–278, 2018. [Online]. Available: https://www.sciencedirect.com/science/article/pii/S1742706118302836 29772347 10.1016/j.actbio.2018.05.014

[39] Jokandan MS , “Bladder wall biomechanics: A comprehensive study on fresh porcine urinary bladder,” J. Mech. Behav. Biomed. Mater, vol. 79, pp. 92–103, 2018. [Online]. Available: https://www.sciencedirect.com/science/article/pii/S1751616117305222 29287227 10.1016/j.jmbbm.2017.11.034

[40] Carrasco JL, King TS, and Chinchilli VM, “The concordance correlation coefficient for repeated measures estimated by variance components,” J. Biopharmaceutical Statist, vol. 19, no. 1, pp. 90–105, 2009, doi: 10.1080/10543400802527890. 19127469

[41] R Core Team, “R: A Language and Environment for Statistical Computing,” R Foundation for Statistical Computing, Vienna, Austria, 2021. [Online]. Available: https://www.R-project.org/

[42] Carrasco JL and Martinez JP, “CCCRM: Concordance correlation coefficient for repeated (and non-repeated) measures,” 2021, r package version 2.0.3, [Online]. Available: https://CRAN.R-project.org/package=cccrm

[43] Damaser MS and Lehman SL, “The effect of urinary bladder shape on its mechanics during filling,” J. Biomech, vol. 28, no. 6, pp. 725–732, 1995. [Online]. Available: http://www.sciencedirect.com/science/article/pii/0021929094001695 7601871 10.1016/0021-9290(94)00169-5

[44] Parekh A. , “Ex vivo deformations of the urinary bladder wall during whole bladder filling: Contributions of extracellular matrix and smooth muscle,” J. Biomech, vol. 43, no. 9, pp. 1708–1716, 2010. [Online]. Available: http://www.sciencedirect.com/science/article/pii/S0021929010001430 20398903 10.1016/j.jbiomech.2010.02.034

[45] Wu B. , “On propagation of axisymmetric waves in pressurized functionally graded elastomeric hollow cylinders,” J. Sound Vib, vol. 421, pp. 17–47, 2018. [Online]. Available: https://www.sciencedirect.com/science/article/pii/S0022460X18300798

[46] Remeniéras J-P , “Acousto-elasticity of transversely isotropic incompressible soft tissues: Characterization of skeletal striated muscle,” Phys. Med. Biol, vol. 66, no. 14, Jul. 2021, Art. no. 145009, doi: 10.1088/1361-6560/ac0f9b. 34186529

[47] Li G-Y and Cao Y, “Assessing the mechanical properties of anisotropic soft tissues using guided wave elastography: Inverse method and numerical experiments,” J. Acoustical Soc. Amer, vol. 142, no. 3, pp. 1526–1536, 2017, doi: 10.1121/1.5002685. 28964064

[48] Kirby R. and Duan W, “Guided wave propagation in cylindrical ducts with elastic walls enclosing a fluid moving with a uniform velocity,” Wave Motion, vol. 85, pp. 1–9, 2019. [Online]. Available: http://www.sciencedirect.com/science/article/pii/S0165212518302300

[49] Pascalis RD, Abrahams ID, and Parnell WJ, “On nonlinear viscoelastic deformations: A reappraisal of Fung’s quasi-linear viscoelastic model,” in Proc. Roy. Soc. A: Math., Phys. Eng. Sci, vol. 470, no. 2166, 2014, Art. no. 20140058, doi: 10.1098/rspa.2014.0058. PMC4042724 24910527

